# What do letters to the editor publish about randomized controlled trials? A cross-sectional study

**DOI:** 10.1186/1756-0500-6-414

**Published:** 2013-10-14

**Authors:** Monika Kastner, Anita Menon, Sharon E Straus, Andreas Laupacis

**Affiliations:** 1 Li Ka Shing Knowledge Institute (LKSKI) of St. Michael’s Hospital, Toronto, ON, Canada; 2 School of Physical and Occupational Therapy, Faculty of Medicine, McGill University, Montreal, QC, Canada; 3 Institute of Health Policy, Management & Evaluation, University of Toronto, Toronto, ON, Canada; 4 Faculty of Medicine, University of Toronto, Toronto, ON, Canada; 5 Keenan Research Centre, LKSKI of St. Michael’s Hospital, Toronto, ON, Canada

**Keywords:** Letters to the editor, Randomized controlled trials, Journalogy

## Abstract

**Background:**

To identify published letters to the editor (LTE) written in response to randomized controlled trials (RCTs), determine the topics addressed in the letters, and to examine if these topics were affected by the characteristics and results of the RCTs.

**Methods:**

Comparative cross-sectional study of a representative sample of RCTs from a set of high-impact medical journals (*BMJ*, *Lancet*, *NEJM*, *JAMA*, and *Annals of Internal Medicine*). RCTs and their published LTE were searched from these 5 journals in 2007. Data were collected on RCTs and their characteristics (author affiliation, funding source, intervention, and effect on the primary outcome) and the topics addressed in published LTE related to these RCTs. Analysis included chi-square and regression analysis (RCT characteristics) and thematic analysis (LTE topics).

**Results:**

Of 334 identified RCTs, 175 trials had at least one LTE. Of these, 381 published LTE were identified. Most RCTs, tested drug interventions (68%), were funded by government (54%) or industry (33%), and described an intervention that had a positive impact on the primary outcome (62%). RCT authors were primarily affiliated with an academic centre (78%). Ninety percent of the 623 LTE topics concerned methodological issues regarding the analysis, intervention, and population in the RCT. There was a significant association between funding source and impact on outcomes (p = 0.002) or type of intervention tested (p = 0.001) in these trials. Clinical and “Other” LTE topics were more likely to be published in response to a government funded RCT (p = 0.005 and p = 0.033, respectively); no other comparisons were significant.

**Conclusions:**

This study showed that most LTE are about methodological topics, but found little evidence to support that these topics are affected by the characteristics or results of the RCTs. The lack of association may be explained by editorial censorship as a small proportion of LTE that are submitted are actually published.

## Background

Published letters to the editor (LTE) are short communications that report study data or more often provide viewpoints that support or challenge a published paper. LTE have also been used to teach critical appraisal skills for medical students [1] and to enhance the educational value of journal clubs [2]. They are considered a form of post-publication peer review and an essential part of scientific debate [3-7]. LTE provide a platform to comment on, clarify and correct published research by alerting readers about aspects of a paper that may have been overlooked by authors, peer-reviewers and editors [5-8]. Some authors have argued that an article is not fully peer-reviewed until after publication [9,10] and that authors of LTE may have more credibility than pre-publication peer reviewers because their opinions are signed, published and thus transparent [3].

However there is some skepticism regarding the representativeness of opinions expressed in published LTE [4-6] (for example, do the statements reflect a general opinion, a clinical practice experience in a given therapeutic area or a suggestion pertaining to the study methods?). Little is known about the topics commonly addressed in LTE and whether these topics are influenced by characteristics of the randomized controlled trial (RCT) (e.g. positive/negative outcomes, presence of serious harm, type of intervention, author affiliation, funding source, etc.). For example, LTE topics may represent opinions that are partially based on whether the RCT reported positive or negative results for the primary outcome. LTE topics may also be influenced by whether an RCT was financially supported by a government body, an academic institution or drug industry. For example, a negative trial of a new drug funded by a peer national granting agency may lead those with a vested interest in the outcome (e.g. company employees, academics whose careers are vested in the drug) to write negative comments about the methodology of the trial, which they might not have done had the trial results been positive. The primary objectives of this empirical study were 1) to identify the types of topics addressed in published LTE for a sample of RCTs, and 2) to examine if these topics were affected by characteristics of the RCTs.

## Methods

Using a quantitative cross-sectional design, we used a representative sample of 5 high impact general medical journals that publish methodologically rigorous RCTs (*BMJ*, *Lancet*, *NEJM*, *JAMA*, and *Annals of Internal Medicine),* to consecutively select RCTs with published LTEs from each journal’s on-line websites (except for rapid responses published by BMJ) for a period of one year beginning with the first issue in January 2006. It was not possible to perform a formal sample size calculation, as there is insufficient evidence to estimate a magnitude of the expected difference in our outcome. However, based on a study with similar methods to investigate the content of LTE [11] (and given that our study was exploratory), we estimated that a sample size of approximately 200 published RCTs with at least one LTE would identify the majority of reasons published LTE are written, and allow for a statistical estimation of the relative frequency of these reasons. We considered only online-published LTE in our sample. To ensure that we captured all published LTE related to their trial to which they pertained, we estimated the publication time period for these letters by analyzing a sample set of 10 RCTs (2 from each journal) and their time to published LTE. We found that responses appeared 3-8 months after the publication of the original RCT, so we selected RCTs published at least one year prior to the beginning of our study period of 2007 (i.e., we searched for RCTs published in 2006). We performed the search for RCTs and their corresponding LTE in PubMed by “AND-ing” each of the 5 journals with the publication year of 2006 (e.g., “Lancet[Jour] AND 2006[pdat]). These yields were then filtered by AND-ing them with the term: “randomized controlled trial”. One reviewer scanned search yields for RCTs with at least one LTE. If no LTE was listed, we entering the RCT citation information in the “single citation matcher” feature of PubMed interface (http://www.ncbi.nlm.nih.gov/pubmed/citmatch) to verify if any correspondence related to the original RCT was available and accessible via hyperlinks.

### Data collection and outcomes

*RCTs:* The following data were extracted from RCTs: the first author and their affiliation, funding source, the characteristics of the intervention (drug, other), and the results (effect upon the primary outcome and harms). Two researchers (MK & AM) abstracted data and classified the trial results independently using a combination of assessment of the benefits and harms and the interpretation of the results as provided and interpreted by the authors of the RCT. RCTs were divided into the following categories: positive impact upon primary outcome (i.e., intervention significantly better than control according to thresholds set by authors) and serious harms or no serious harms; negative impact upon the primary outcome (i.e., intervention is significantly worse than control); no impact upon the primary outcome (i.e., intervention neither better nor worse than control) and serious harms or no serious harms. Harms were recorded based on what authors reported in the results and discussion section of their paper. The purpose of this investigation was to identify any possible associations between the outcomes of the RCT and the topic(s) of the LTE that was written in response.

*Letters to the Editor:* We extracted data from published LTE including their topics and objectives, and how these themes related to the design and results of the RCT. Three broad topic categories were identified *a priori* by the research team: Methodological (topics related to the RCT’s study design, outcomes, population, intervention, and analysis); clinical (topics describing biological mechanisms, diagnosis, and treatment of the clinical area); and “other” (any aspect of the RCT not related to methodological or clinical topics).

### Analysis

Descriptive statistics were used to determine 5 RCT characteristics: impact on primary outcomes (positive, negative, none), presence of serious harms (harm, no harm), type of intervention tested (drug, other), author affiliation (i.e., academic centre, hospital, research institute, community practice/clinic, government, and industry), and funding source (government, academic, industry, other, not indicated). We also investigated the proportion of LTE by topic category within each of the 5 journals. RCT-level comparisons were analyzed using chi-square statistics to test associations between the impact on outcomes reported in the RCT (positive/negative/none) and its funding source; and the type of intervention tested in the RCT (drug/other) and its funding source using SPSS, version 17.0. All 5 RCT characteristics were considered of similar importance in the analysis. To account for RCTs with different numbers of LTE, we used Poisson regression analysis to test LTE-level comparisons. We tested associations between LTE topic and the following RCT characteristics: impact of the intervention on primary outcomes, presence of serious harm, type of intervention, funding source, and author affiliation. LTE topics were analyzed qualitatively using thematic analysis, which involved two researchers (MK and AM) independently classifying topics into 3 broad categories (methodological, clinical, other), and then iteratively identifying sub-categories and themes within these core categories. Two researchers (AL, SS) were consulted to resolve conflicts, and to verify the appropriateness and relevance of the final topic classifications and their labels.

## Results

Searches in the 5 journals for the year 2007 identified 334 RCTs, of which175 trials had at least one published LTE, generating a total of 381 published LTE (range 1-5 LTE per RCT) (Table 1). The *NEJM* had the highest yield of RCTs (54%), LTE (61%), and topics (58%) followed by *JAMA*, Lancet, *BMJ*, and *Ann Int Med*. Of the 175 RCTs, 62% reported interventions that had a positive impact on the primary outcome, and 92% reported no statistically significant increase in adverse events related to the intervention (as indicated by study authors). Most RCTs (68%) were drug interventions, 54% of which were funded by government and 33% by industry. Authors of the RCT were primarily affiliated with an academic centre (78%).

**Table 1:** Proportion of RCTs, LTE, and categories of LTE themes by journal*

**Journal**	**Number of RCTs (%)**	**Number of LTEs (%)**	**Number of topics (%)**	**Categories of LTE topics (N = 623) (%)**		
				**Methodological**	**Clinical**	**Other**
NEJM	94 (54)	233 (61)	359 (58)	325 (58)	26 (62)	8 (47)
JAMA	35 (20)	56 (15)	108 (17)	107 (19)	0 (0)	1 (6)
Lancet	27 (15)	60 (16)	110 (18)	95 (17)	7 (17)	8 (47)
BMJ	11 (6)	19 (5)	26 (4)	18 (3)	8 (19)	0 (0)
AnnIntMed	8 (5)	13 (3)	20 (3)	19 (3)	1 (2)	0 (0)
TOTAL	175	381	623	564 (91)	42 (7)	17 (3)

*RCT = randomized controlled trial; LTE = letter to the editor.

**Figure 1:**
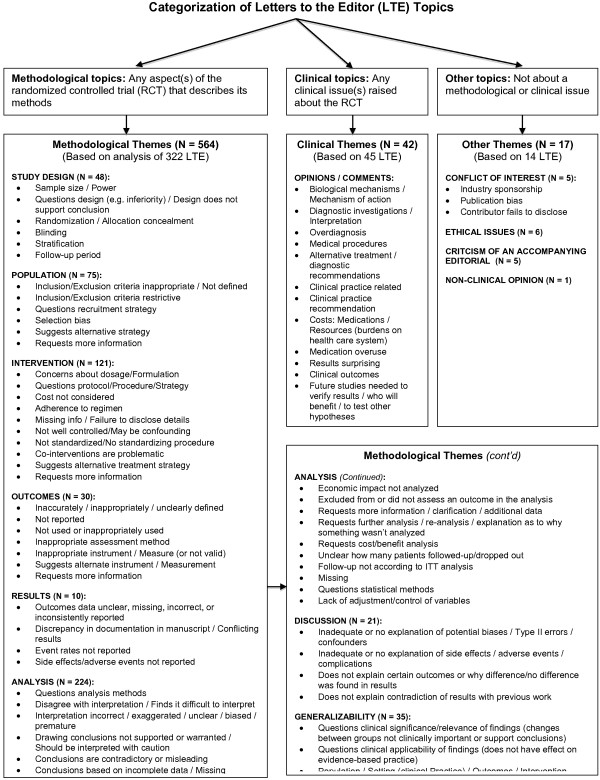
**Classification of LTE topics according to three broad categories *(Methodological, Clinical, Other).***

Of 381 published LTEs, 623 topics were identified (range 1-5 topics per LTE)—564 (90%) methodological, 42 (7%) clinical and 17 (3%) other. The Figure shows how these topic themes were categorized. Topics in the methodological theme were grouped into a number of categories such as statistical analysis, results, discussion, and generalizability (Figure 1). Clinical topics represented opinions or comments about medical procedures, clinical practice issues, costs (e.g., medications, resources), clinical outcomes, and topics related to “surprising” or unexpected results or recommendations for conducting future studies to verify results. Topics in the “Other” category comprised conflict of interest (i.e., industry sponsorship, publication bias, and failure of contributor(s) to disclose information), ethical issues, criticisms of an accompanying editorial, and non-clinical opinions.

Ninety percent of the topics discussed in LTE were methodological; most addressed the analysis (40%), intervention (21%), and population (13%) (Table 1). Sixty-one percent of letters about clinical themes were about clinical opinions (e.g., *“A double standard clearly exists among the screening community, who seem to be in denial - the problem of over-diagnosis that was finally confirmed by irrefutable evidence in the study can no longer be denied”*). In the “Other” category, 41% of LTE topics were related to ethical issues (e.g., *“Study patients should not pay for trial medications and we are surprised that the local ethics committee ignored this fact”*); 29% were about conflict of interest (e.g., *“One of the principal contributors fails to declare a given conflict of interest which can be found in a Cochrane review”*); and 29% were about a criticism of an accompanying editorial (e.g., *“…The statement in the accompanying editorial that the results of the author are not likely to influence current clinical practice is perplexing”*).

### RCT-level comparisons

A significant association between funding source and the impact of the intervention on outcomes in the RCTs (p = 0.002) was found. Of 108 RCTs with a positive impact on outcomes, 44% were funded by government, 40% by industry, 7% by academic, and 4% by other (5% did not indicate); “no impact” RCTs (N = 58) were primarily funded by government (78%), while about half of the negative impact trials (N = 9) were funded by industry (56%). There was also a statistically significant association between funding source and the type of intervention (p = 0.001). Most drug intervention RCTs (N = 119) were funded by government (49%) or industry (42%), while “other” intervention RCTs (N = 56) was funded mostly by government (66%). Industry-funded RCTs (N = 56) tested more drug interventions than government-funded RCTs (N = 95) (89% vs 61%; p = 0.001 [Pearson Chi-square 19.86]). No statistically significant associations were found for other RCT-level comparisons such as impact of primary outcome or type of intervention tested and author affiliation. Please see Additional file 1: Table S1 for additional data on the proportion of RCTs according to impact on primary outcome and type of intervention by funding source.

### LTE-level comparisons

Of the LTE addressing methodological topics, 54% were in response to government-funded RCTs and 32% responded to industry-funded RCTs. Letters about clinical or other topics were mostly about RCTs funded by government (55%, 52%, respectively). Poisson regression analysis showed a statistically significant association between RCT funding source and topic of the LTE (p = 0.002), indicating that clinical and “other” LTE topics were more likely to be published in response to a government funded RCT (p = 0.005 and p = 0.033, respectively). Table 2 shows the differences between the remainder of RCT characteristics (impact on outcome, presence of serious harm, type of intervention, and author affiliation) and LTE topics—none of these were significant. Please see Additional file 1: Table S2 for additional data on the proportion of LTE themes by funding source of the RCT.

**Table 2:** RCT characteristics and LTE-level comparisons*

**RCT characteristic**	**Number of Topics (%)**			**P-value†*RCT characteristic compared with LTE topic: Methodological, Clinical or Other***
**(Number of RCTs; N = 175)**	**Method (N = 564)**	**Clinical (N = 42)**	**Other (N = 17)**	
Impact on outcomes reported in the RCT				0.581
Positive (N = 108)	348 (62)	29 (69)	13 (76)	
Negative (N = 9)	28 (5)	5 (12)	0 (0)	
None (N = 58)	188 (33)	8 (19)	4 (24)	
Presence of serious harm in outcomes of the RCT				0.770
Harm (N = 161)	502 (89)	36 (86)	16 (94)	
No harm (N = 4)	62 (11)	6 (14)	1 (6)	
Type of intervention tested in the RCT				0.917
Drug (N = 119)	392 (70)	27 (64)	12 (71)	
Other (N = 56)	172 (30)	15 (36)	5 (29)	
Author affiliation of the RCT				0.487
Academic (N = 137)	438 (78)	32 (76)	9 (53)	
Hospital (N = 26)	94 (17)	7 (17)	7 (41)	
Research Institute (N = 6)	15 (2)	1 (2)	0 (0)	
Community practice (N = 3)	10 (1.8)	0 (0)	1 (6)	
Government (N = 2)	6 (1)	0 (0)	0 (0)	
Industry (N = 1)	1 (0.2)	2 (5)	0 (0)	
Funding source of the RCT				0.002‡
Government (N = 95)	303 (54)	23 (55)	9 (53)	
Academic (N = 9)	30 (5)	3 (7)	0 (0)	
Industry (N = 56)	178 (32)	6 (14)	4 (24)	
Other (N =7)	12 (2)	4 (10)	0 (0)	
Not indicated (N = 8)	41 (7)	6 (14)	4 (24)	

*RCT = randomized controlled trial; Method. = Methodological; LTE = letter to the editor.

†Calculated using Poisson Regression analysis.

‡Significant at p < 0.05.

## Discussion

We conducted a descriptive study to identify topics addressed in LTEs of published RCTs, and whether these topics may be affected by characteristics of the RCTs to which they refer. Analysis of 175 RCTs with corresponding LTEs that were published in five journals showed that the majority of RCTs reported a positive impact on the primary outcome, with 38% reporting no impact or negative impact on primary outcomes. The majority of published LTE focused on methodological aspects of the RCT specific to its analysis, intervention and population (Table 3). These results support the idea that LTEs can be used as a form of post-publication peer review and that they can play an important role in identifying potential methodological flaws [7,12]. However, only a small proportion of articles undergo substantive criticism in LTE (e.g., a problem that suggest fatal flaws in the design that could invalidate the research) [4,12]. Gotzsche et al found that although 88% of rapid responses were available for research papers submitted to the BMJ, only 30% raised substantive criticism, of which only 19% were published in the print version of the journal [4]. Baethge et al conducted a search in Medline in 2007, and found that only 15,312 letters and comments were generated from a total of 117,843 original published clinical articles and reviews [12]. Furthermore, criticisms are often ignored and undervalued by researchers and clinicians even though they have the potential to shape clinical knowledge [4,6,7]. This work also highlights the potential for LTE to be used as a unique knowledge translation tool for journal readers (clinicians and researchers) and editors. For example, LTE could be used as a platform to advance knowledge around research methods; clinical gaps and clinical experiences (i.e., what works or doesn’t work under different circumstances); and to provide the opportunity to share/suggest ideas, research questions or next steps by fostering collaborations across research groups (and continents) to minimize redundancies and duplication of efforts.

**Table 3:** Number of letters according to sub-categories of methodological themes by journal

**Journal**	**Study design**	**Outcomes**	**Population**	**Intervention**	**Analysis**	**Results**	**Discussion**	**Generalizability**	**TOTAL**
NEJM	30	14	50	72	130	5	9	15	325 (58)
JAMA	9	6	15	19	46	0	2	10	107 (19)
Lancet	6	6	10	19	38	4	6	6	95 (17)
BMJ	1	3	0	3	7	1	1	2	18 (3)
AnnIntMed	2	1	0	8	4	0	3	1	19 (3)
TOTAL	48 (9)	30 (5)	75 (13)	121 (21)	225 (40)	10 (2)	21 (4)	34 (6)	564

To our knowledge, this is the first study to investigate the types of topics addressed in published LTE for a sample of RCTs across 5 high impact journals, and to test the association between the outcomes of the RCT and the topic(s) of the LTE that was written in response to the RCT. There are some limitations to this study. We searched only 5 medical journals, so our sample size may have been too small to detect significant differences. However, we included the journals that publish a large proportion of methodologically rigorous randomized trials, which likely maximized the number of LTE and the breadth (reaching researchers as well as clinicians) and depth (more focused and detailed discussions) of topics. Our focus on high-impact journals may have also biased the ‘type’ of scrutiny provided by their letters compared with those published in lower impact journals—an important consideration if LTE are used for the purposes of post-publication peer review. Indeed, LTE addressing methodological topics might be more often reported in lower impact journals. In addition, not all letters submitted to these journals get published, and we did not account for the balance of RCTs published across journals (i.e., some publish more trials per issue than others), so the frequency of topics we identified may not be an accurate representation of all the issues raised by LTE. Furthermore, we did not compare RCT characteristics between studies that generated a letter with those that did not – this information may have strengthened our findings and is a potential topic for a future study. Another limitation is that we may have also missed some letters that could have been published beyond our predicted 1-year period. However, it is unlikely that a small number of additional letters would have influenced our findings. Finally, analysis of all letters submitted to journals (not just the ones published by journals or those accessible electronically) may have also increased our power to detect differences.

## Conclusions

The study found little evidence that LTE topics are affected by the characteristics or results of their corresponding RCTs. However, some letters that are submitted do not get published, so these findings may in part reflect editorial censorship as journal editors make the final decision on LTE publications. Our findings also showed that most LTEs from 5 high-impact medical journals are about methodological topics, which may indicate a potential for LTE to be used as a form of post-publication peer review.

## Competing interests

The authors declare that they have no competing interests.

## Authors’ contributions

AL and MK designed the study protocol. MK and AM collected and analyzed the data and developed the LTE classification of topics and themes. AL and SS resolved conflicts, and verified the clinical appropriateness and relevance of the final topic classifications and their labels. MK and AM prepared the initial manuscript. All authors commented on the final manuscript before submission. All authors read and approved the final manuscript.

## Supplementary Material

Additional file 1: Table S1 Proportion of RCTs according to impact on primary outcome and type of intervention by funding source and **Table S2.** Proportion of LTE themes by funding source of the RCT*. Click here for file
