# Supplementary material for: What do letters to the editor publish about randomized controlled trials? A cross-sectional study
Source: BMC Res Notes. 2013 Oct 14;6:414. doi: 10.1186/1756-0500-6-414 (PMC3852599; doi:10.1186/1756-0500-6-414)
Supplement: Additional file 1: Table S1 — Proportion of RCTs according to impact on primary outcome and type of intervention by funding source and Table S2. Proportion of LTE themes by funding source of the RCT*. [file 1756-0500-6-414-S1.doc]

**Additional file 1**

Table S1. Proportion of RCTs according to impact on primary outcome and type of intervention by funding source

| **Funding source** | **Number of RCTs (%)** | **Number of RCTs according to impact on primary outcome (%)** | | | **Number of RCTs according to type of intervention** | |
| --- | --- | --- | --- | --- | --- | --- |
|  |  | Positive | Negative | No | Drug | Other |
| Government | 95 (54) | 48 (44) | 2 (22) | 45 (78) | 58 (49) | 37 (66) |
| Academic | 9 (5) | 8 (7) | 0 (0) | 1 (2) | 5 (4) | 4 (7) |
| Industry | 56 (32) | 43 (40) | 5 (56) | 8 (14) | 50 (42) | 6 (11) |
| Other | 7 (4) | 5 (5) | 1 (11) | 1 (2) | 3 (2) | 4 (7) |
| Not Indicated | 8 (5) | 4 (4) | 1 (11) | 3 (5) | 3 (2) | 5 (9) |
| **TOTAL** | **175** | **108 (62)** | **9 (5)** | **58 (33)** | **119 (68)** | **56 (32)** |

**Additional file 1**

Table S2. Proportion of LTE themes by funding source of the RCT*

| **Funding source** | **Number of RCTs** | **Number of LTE topics (%)** | **LTE topic theme** | | |
| --- | --- | --- | --- | --- | --- |
|  |  |  | Methodological (%) | Clinical (%) | Other (%) |
| Government | 94 (54) | 335 (54) | 303 (54) | 23 (55) | 9 (52) |
| Academic | 9 (5) | 33 (5) | 30 (5) | 3 (7) | 0 (0) |
| Industry | 57 (32) | 186 (30) | 178 (32) | 6 (14) | 4 (24) |
| Other | 7 (4) | 12 (2) | 12 (2) | 4 (10) | 0 (0) |
| Not Indicated | 8 (5) | 57 (9) | 41 (7) | 6 (14) | 4 (24) |
| **TOTAL** | **175** | **623** | **564 (91)** | **42 (7)** | **17 (3)** |

*LTE = letter to the editor; RCT = randomized controlled trial.
